# Biophysical and economic implications for agriculture of +1.5° and +2.0°C global warming using AgMIP Coordinated Global and Regional Assessments

**DOI:** 10.3354/cr01520

**Published:** 2018-09-04

**Authors:** Alex C. Ruane, John Antle, Joshua Elliott, Christian Folberth, Gerrit Hoogenboom, Daniel Mason-D’Croz, Christoph Müller, Cheryl Porter, Meridel M. Phillips, Rubi M. Raymundo, Ronald Sands, Roberto O. Valdivia, Jeffrey W. White, Keith Wiebe, Cynthia Rosenzweig

**Affiliations:** 1 NASA Goddard Institute for Space Studies, New York, NY 10025, USA; 2 Oregon State University, Corvallis, OR 97331, USA; 3 University of Chicago, Chicago, IL 60637, USA; 4 International Institute for Applied Systems Analysis, 2361 Laxenburg, Austria; 5 University of Florida, Gainesville, FL 32611, USA; 6 International Food Policy Research Institute, Washington, DC 20005, USA; 7 Commonwealth Science and Industrial Research Organisation, St Lucia, QLD 4067, Australia; 8 Potsdam Institute for Climate Impacts Research, 14473 Potsdam, Germany; 9 Columbia University Center for Climate Systems Research, New York, NY 10025, USA; 10 USDA Economic Research Service, Washington, DC 20036, USA; 11 USDA Agricultural Research Service, Maricopa, AZ 85239, USA

**Keywords:** Climate change, Agricultural system, Food prices, Mitigation, CO_2_, Crop model, Climate stabilization, Climate impact

## Abstract

This study presents results of the Agricultural Model Intercomparison and Improvement Project (AgMIP) Coordinated Global and Regional Assessments (CGRA) of +1.5° and +2.0°C global warming above pre-industrial conditions. This first CGRA application provides multi-discipline, multi-scale, and multi-model perspectives to elucidate major challenges for the agricultural sector caused by direct biophysical impacts of climate changes as well as ramifications of associated mitigation strategies. Agriculture in both target climate stabilizations is characterized by differential impacts across regions and farming systems, with tropical maize *Zea mays* experiencing the largest losses, while soy *Glycine max* mostly benefits. The result is upward pressure on prices and area expansion for maize and wheat *Triticum aestivum*, while soy prices and area decline (results for rice *Oryza sativa* are mixed). An example global mitigation strategy encouraging bioenergy expansion is more disruptive to land use and crop prices than the climate change impacts alone, even in the +2.0°C scenario which has a larger climate signal and lower mitigation requirement than the +1.5°C scenario. Coordinated assessments reveal that direct biophysical and economic impacts can be substantially larger for regional farming systems than global production changes. Regional farmers can buffer negative effects or take advantage of new opportunities via mitigation incentives and farm management technologies. Primary uncertainties in the CGRA framework include the extent of CO_2_ benefits for diverse agricultural systems in crop models, as simulations without CO_2_ benefits show widespread production losses that raise prices and expand agricultural area.

## 1. INTRODUCTION

Signatures of climate change are already evident in observations of natural and human systems, and the continuing rise of world greenhouse gas emissions suggests that society will face substantially altered climate conditions in the future (IPCC 2013). The extent of climate change will be determined by societal activities that result in the overall burden of greenhouse gas emissions and land use changes, as will the relative shares of mitigation, adaptation, and impact that will characterize the emergent climate equilibrium (IPCC 2014a,b,c). Climate policy could therefore be oriented toward striking a balance to avoid both the highest costs of mitigation (to keep climate change low) and the highest burden on adaptation and unavoidable climate impacts (when climate change is high) (IPCC 2014c, O’Neill et al. 2017b). Representatives from 196 countries signed the United Nations Framework Convention on Climate Change (UNFCCC) Paris Agreement (UNFCCC 2015) in December 2015 aiming for such a balance, setting a goal to limit global mean temperature rise below 2°C above pre-industrial levels, with nationally determined commitments aiming to reach a stabilization at +1.5°C above pre-industrial conditions.

This study focuses on the agricultural sector impacts of global warming at the limits of these ambitious mitigation targets, defining a ‘+1.5°C World’ and ‘+2.0°C World’ (relative to pre-industrial conditions) and assessing the biophysical and economic implications from local to global scales. This multidisciplinary and multi-scale perspective is essential, given our increasingly complex and interconnected agricultural systems, wherein farm outputs are traded in local, regional, and global markets that set prices motivating farmer decisions and practices in agricultural systems around the world. Assessment of future climate challenges must also recognize shifts in agricultural technology, socioeconomic development, dietary demand, and international policies that will shape any future world.

The Agricultural Model Intercomparison and Improvement Project (AgMIP; Rosenzweig et al. 2013, 2015) was launched in 2010 to provide systematic approaches capable of modeling these shifts in future agricultural food systems. AgMIP links agricultural communities, scientific approaches, and models for climate, crops, livestock, economics, nutrition, and food security responses. AgMIP protocol-based studies of various crop and livestock species, spatial scales, and models provide a basis for integrated assessment, multi-sectoral analysis, and scenario application (Ruane et al. 2017). Prior studies have focused largely on agricultural impacts of climate changes beyond +2.0°C (IPCC 2013, Rosenzweig et al. 2014, Wiebe et al. 2015), but the impact of high mitigation scenarios such as the +1.5° and +2.0°C Worlds has received relatively little attention.

**Fig. 1.**
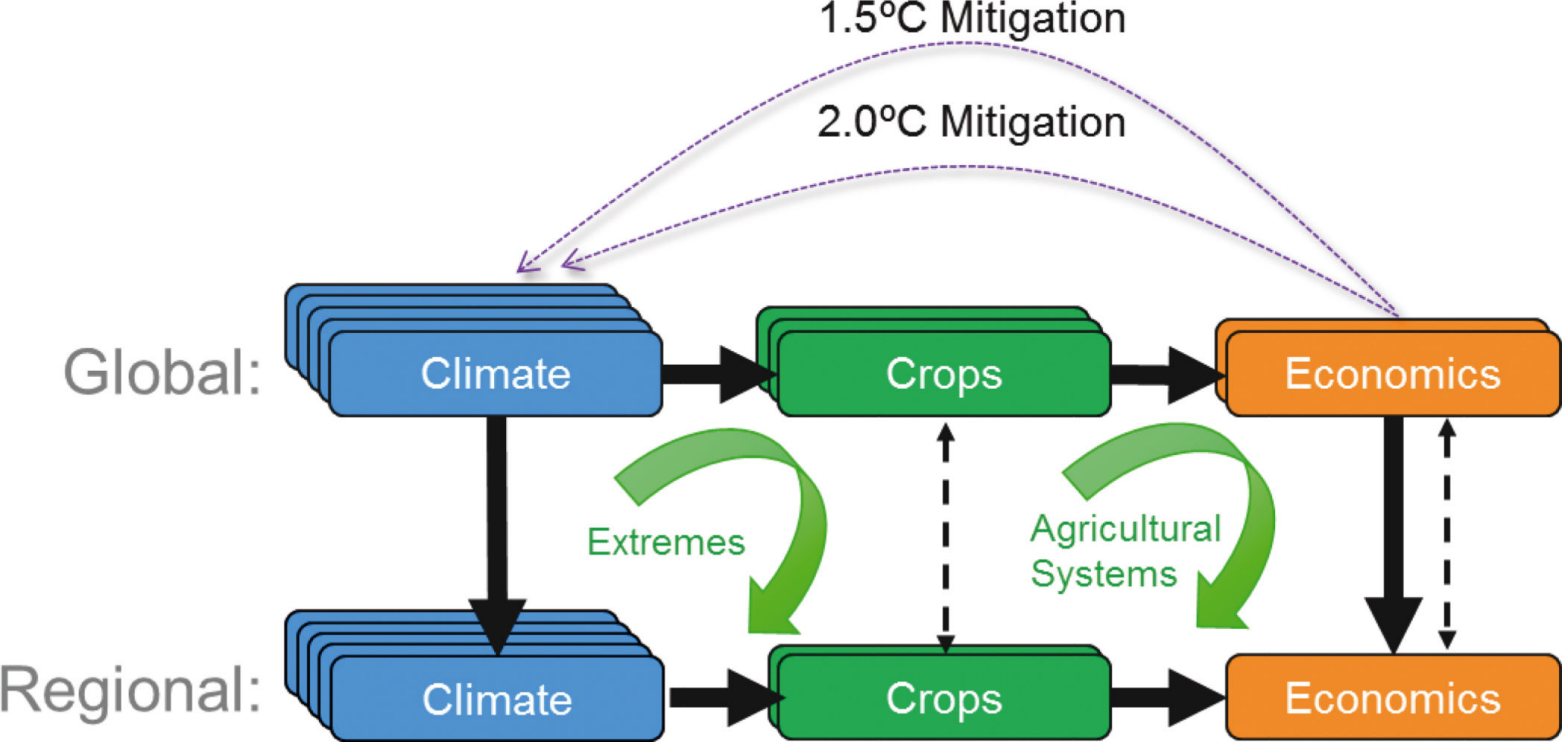
Coordinated Global and Regional Assessments (CGRA) linking global and regional scales, disciplines, and multiple models with a focus on +1.5° and +2.0°C warming worlds. Extreme events and alternative agricultural systems for adaptation and mitigation are also explored on the nexus of disciplines and scales. Solid lines indicate direct use of model outputs as inputs for successive modeling in the core CGRA application, while dashed lines indicate cross-scale comparisons enabled. Mitigation scenarios examine potential policy and socioeconomic development pathways that would limit cumulative greenhouse gas emissions and determine resulting climate stabilizations. The CGRA also enables multi-perspective analysis of the agricultural sector impacts of extreme events and the resilience of alternate future agricultural systems

To explore agricultural conditions in the +1.5° and +2.0°C Worlds, we employed AgMIP’s Coordinated Global and Regional Assessments (CGRA) framework (Rosenzweig et al. 2016). CGRA links across agricultural models, disciplines, and spatial scales, using common scenario assumptions and a harmonizing model output/input framework to elucidate interactions that may be overlooked in isolated studies (Fig. 1). Given the urgency within the UNFCCC community for scientific insights into the implications of +1.5° and +2.0°C global warming, here we present the results of a fast-track assessment of the AgMIP CGRA designed to capture key responses and messages. Rosenzweig et al. (2018) laid out the concept of this +1.5° and +2.0°C global warming assessment, and here we present the full multi-discipline, multi-model, and multi-scale results. Future augmentation could examine additional feedback loops, participating models, regional case study perspectives, and scenario combinations focused on land use, climate challenges, socioeconomic development, consumption patterns, and management trade-offs.

CGRA assessments of the +1.5° and +2.0°C Worlds include a core set of directly connected models and analyses (presented below), as well as a series of linked studies using common scenarios, assumptions, and modeling frameworks to facilitate coordinated analyses (Rosenzweig et al. 2018). Diverse regional case studies provide unique perspectives that would be missing from top-down global app roaches; however, these are not meant to comprehensively represent the many farming systems and populations that constitute the global agricultural sector. Table 1 describes the overall set of models used in the core CGRA study. Global climate scenarios and challenges for agricultural regions are described in Section 2 and detailed in Ruane et al. (2018). Global crop production simulations are presented in Section 3. Global economic model results project market impacts of climate changes and mitigation policies in Section 4, while Section 5 examines more detailed case studies of biophysical impact and regional integrated assessments for farm population economics in Pakistan and the US (with additional analyses provided by Antle et al. 2018). Linked studies provide enhanced +1.5° and +2.0°C World detail on agricultural trade and integrated assessment model mitigation pathways (van Meijl et al. 2018), food security implications of mitigation efforts (Hasegawa et al. 2018), the changing nature of extreme climate events and uncertainty related to CO_2_ effects (Schleussner et al. 2018), and enhanced regional analyses for Europe (H. Webber pers. comm.) and West Africa (Faye et al. 2018). We conclude with a discussion of major messages and priorities for CGRA development and application.

## 2. CLIMATE CHANGES FOR AGRICULTURAL REGIONS

Future worlds examined in this study are defined by a new climate stabilization where global mean surface temperatures are +1.5° or +2.0°C above preindustrial conditions. This involves defining the preindustrial period and time horizon of climate stabilizations, and then exploring projected impacts of the embedded shifts in regional climate patterns, seasonality, and extreme conditions that will affect agricultural systems. Climate scenario generation and agro-climatic analysis for the CGRA +1.5° and +2.0°C study is detailed in Ruane et al. (2018) and summarized below.

### 2.1. Representing +1.5° and +2.0°C World climates

Understanding of future and alternate climate states comes primarily from the outputs of global climate models (GCMs) from earth system modeling groups participating in the Coupled Model Intercomparison Project (CMIP; Taylor et al. 2012, Eyring et al. 2016). In CMIP5, future projections took the form of transient simulations driven by representative concentration pathways (RCPs; Moss et al. 2010), providing outputs from more than 30 modeling groups, but no clear projection of a +1.5° or +2.0°C stabilized climate state.

The Half a degree Additional warming, Projections, Prognosis and Impacts project (HAPPI; Mitchell et al. 2017) took on the challenge of estimating these stabilized worlds, and thus HAPPI outputs form the primary climate projections for this study. HAPPI established climate drivers for the +1.5°C World by drawing from conditions at the end of the 21st century within RCP2.6 (e.g. greenhouse gas and aerosol concentrations, land use, and sea surface temperature anomalies) and combined RCP2.6 and RCP4.5 for the +2.0°C World. HAPPI defines the pre-industrial period as 1860–1880, a relatively stable climate period absent major volcanic eruptions at the beginning of the modern meteorological station record. GCMs participating in HAPPI then conducted initial condition ensembles to examine natural variability and extreme characteristics of the 2006–2015 period (‘current climate’), then drove ensemble simulations mimicking stabilized +1.5° and +2.0°C Worlds pegged to the 2106–2115 period. As the current climate period (~2010) is already ~1°C above preindustrial conditions, the +1.5° and +2.0°C Worlds require an additional ~0.5 and 1°C of global warming (Morice et al. 2012). Future world simulations maintain a degree of uncertainty around the desired global mean surface temperature increase, given differences in the transient climate responses of GCMs to imposed forcings (MIROC5, in particular, was noted as being warmer than expected). Ruane et al. (2018) further described how these uncertainties may affect agro-climatic scenarios, and also compared the HAPPI subset of GCMs against climate conditions simulated when the RCP transient simulations cross the +1.5° and +2.0°C thresholds. In general, largely similar global conditions are present in both CMIP transients and HAPPI stabilization scenarios, but HAPPI produces warmer conditions over the rice-growing areas of Asia, owing to its use of cleaner end-of-century RCP2.6 tropospheric aerosol concentrations, while most CMIP transients cross +1.5° and +2.0°C global warming earlier in the 21st century.

Climate scenarios for maize *Zea mays*, wheat *Triticum aestivum*, rice *Oryza sativa*, and soy *Glycine max* seasons focus on months between planting and harvest (according to the AgMIP Global Gridded Crop Model Intercomparison protocols, GGCMI; Elliott et al. 2015). Wheat-growing areas match the primary spring or winter wheat-growing season according to GGCMI simulated yields, with climate scenarios capturing the final 90 d of winter wheat before harvest in order to avoid the dormant vernalization period following planting (as in Ruane et al. 2018). Climate changes (mean maximum and minimum temperatures, mean precipitation, the number of wet days, and the standard deviation of daily maximum and minimum temperatures) were calculated for each month from the HAPPI ensemble for each GCM (Table 1). While HAPPI provides climate changes from a ~2010 current period climate, AgMIP’s GGCMI and local crop modeling protocols utilize a 1980–2009 ‘recent observed climate’ as the baseline, necessitating a simplified pattern-scaling estimation of climate changes between these different baseline climates (based upon local changes per degree of global temperature change in the HAPPI +1.5°C World simulation; see Ruane et al. 2018). CO_2_ concentrations recommended by HAPPI for the +1.5°C World (423 ppm) and +2.0°C World (487 ppm) are higher than many transient simulations at the same global temperature threshold, although the CO_2_ concentration in any climate stabilization depends on a climate model’s climate sensitivity (Ruane et al. 2018). Together with climate changes aggregated over the growing season, these provide the driving conditions for global crop model yield estimates, and monthly changes are imposed on local weather observations to create daily time series scenarios for local crop model simulation using the mean-and-variability change ‘enhanced delta’ approach described by Ruane et al. (2015a).

### 2.2. Climate projections for agricultural regions

**Fig. 2.**
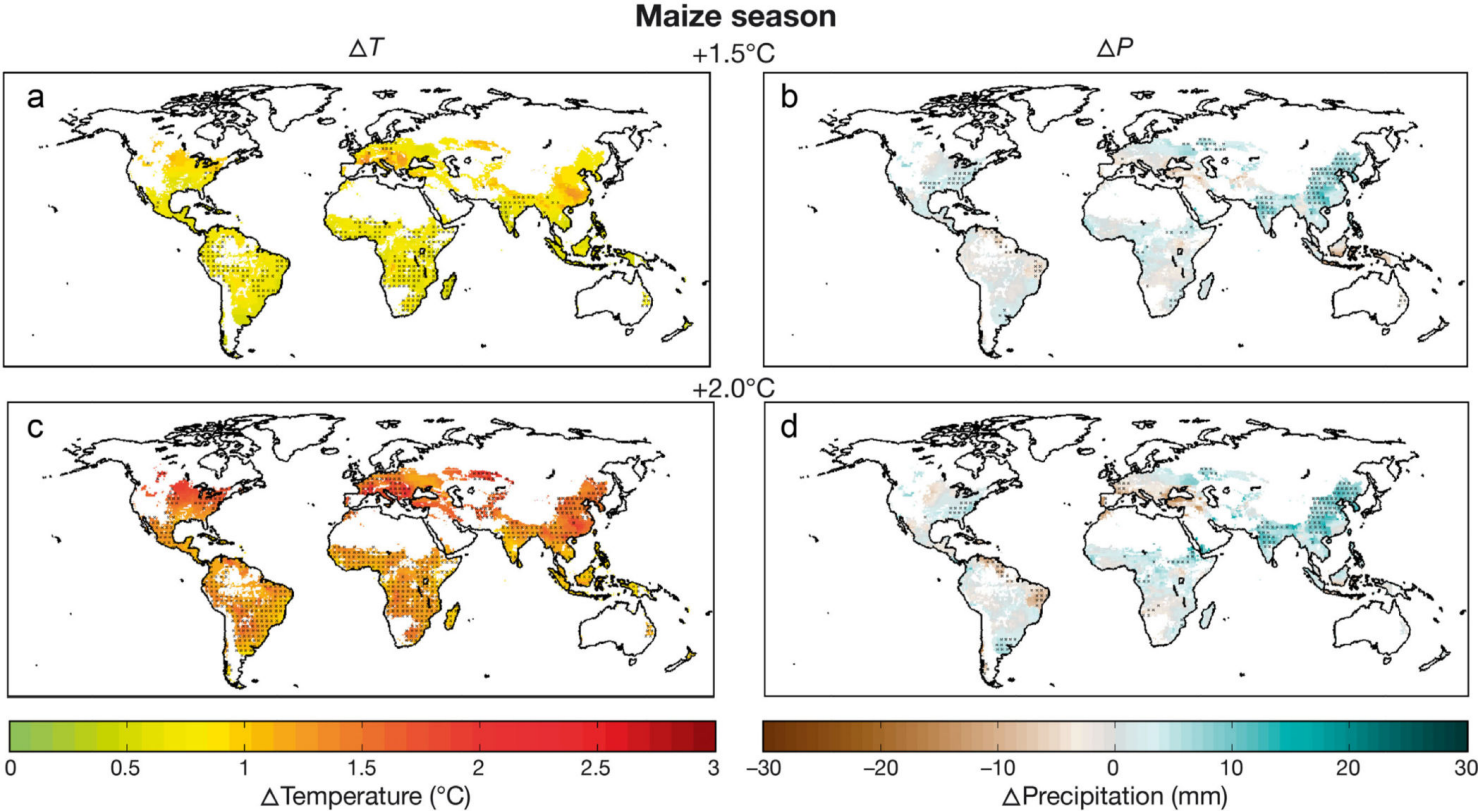
Rainfed maize season median (a,c) temperature and (b,d) precipitation changes for the +1.5°C World (a,b) and +2.0°C World (c,d); HAPPI simulations compared to current period (~2010) climate. Hatch marks for temperature indicate that median changes are larger than twice the range across global climate models and signal agreement in 4 out of the 5 HAPPI models for the direction of mean precipitation change. Scenarios were generated for all regions, but only grid cells with >10 ha are presented to highlight substantial production regions (You et al. 2014)

HAPPI Climate changes for the +1.5° and +2.0°C Worlds contain many of the same patterns observed in recent IPCC assessments (Collins et al. 2013), including warming that exceeds the global average over land (due to the ocean’s higher heat capacity) at higher latitudes (owing to local feedbacks), and in the winter season. Global precipitation rises slightly as global temperatures increase, but this effect is small compared to regional shifts in mean precipitation that largely track an exacerbation of moisture convergence and divergence regions associated with global warming’s enhancement of the hydrologic cycle. Fig. 2 presents median rainfed maize season projections for the +1.5° and +2.0°C Worlds compared to the current (~2010) climate, showing a pace of robust warming that exceeds global mean temperature rise for nearly all maize-growing regions and additional warming at higher latitudes and over portions of the East Asian monsoon (due in part to assumed aerosol policies). Median warming does not exceed twice the range among GCMs in many mid-latitude regions until the +2.0°C scenario or beyond, while the signal more readily emerges above relatively consistent projections in the Tropics. Precipitation changes are largely uncertain across models in the +1.5°C World, although patterns strengthen somewhat under the warmer +2.0°C World. Wetter conditions are notable in the Asian monsoon region, southeastern US, and the lower Rio de la Plata basin, while drier conditions are projected for southern Europe and northeastern South America. Ruane et al. (2018) detailed projections for additional growing seasons examined in the CGRA assessments, as well as the tendency of many growing regions to face more extreme interannual variability under the +1.5° and +2.0°C Worlds. Rosenzweig et al. (2018) provided a further exploration of GCM uncertainty for the rainfed wheat season.

## 3. AGRICULTURAL SYSTEM RESPONSES TO CLIMATE CHANGES

Climate shifts associated with the +1.5° and +2.0°C World will affect cereal production around the world, with impacts dependent on the farming system environment (soils and baseline climate), cultivar selection, and agricultural management. The AgMIP GGCMI utilizes partially harmonized inputs as well as common protocols and output processing pipel ines to facilitate multi-model simulation of agricultural production with global coverage and ½° × ½° horizontal resolution (Elliott et al. 2015). GGCMI provided longterm agricultural production impact projections under various CMIP5 RCPs (Rosenzweig et al. 2014) and recently completed a historical period intercomparison and benchmark evaluation against observed yields to elucidate model strengths and uncertainties (Müller et al. 2017). GGCMI models are configured to capture direct weather and climate responses but do not simulate additional factors that may affect seasonal variability and long-term outlooks (e.g. pests, diseases, weeds, river flooding, ozone).

### 3.1. Simulating +1.5° and +2.0°C World agricultural production

Agricultural production in the +1.5° and +2.0°C Worlds was projected using outputs from GGCMI Phase 2, a systematic sensitivity test exploring responses to regional changes in CO_2_, temperature, water, nitrogen, and adaptation (Elliott et al. 2015, Ruane et al. 2017). GGCMI models were first run over the 1980–2009 period climate (provided by AgMERRA; Ruane et al. 2015b), and then executed under a range of imposed mean changes in CO_2_ (360 to 810 ppm), temperature (−1 to +6 °C), water (−50 to +30% precipitation change), nitrogen fertilizer (10 to 200 kg ha^−1^), and cultivar adaptation (with or without cultivars selected to maintain growing season length). Sensitivity tests were run in isolation and in combination, providing a sampling of the climate change space capturing the climate changes projected for the +1.5° and +2.0°C Worlds at CO_2_ levels of 423 and 487 ppm, respectively.

Yield levels for the HAPPI scenarios (current period, +1.5°C World, and +2.0°C World) were estimated from GGCMI Phase 2 outputs using the HAPPI seasonal climate scenarios (providing changes in temperature, water, and CO_2_) and holding farm system management constant (no change in N, planting dates, or cultivar adaptation). Outputs from 3 global gridded crop models (GGCMs) were used for the CGRA study (see Table 1 and additional de tails in the Supplement at www.int-res.com/ articles/suppl/c076p017_supp.pdf). Here we employ crop simulations provided by the GGCMs GEPIC (Folberth et al. 2012), LPJmL (von Bloh et al. 2018, and pDSSAT (Elliott et al. 2014). GGCM projections are driven by mean local climate changes; however, these interact with daily and seasonal events and alter extreme events that affect total yield levels (see Schleussner et al. 2018, for a further examination of yield extremes in the +1.5° and +2.0°C Worlds).

### 3.2. Agricultural production change projections

**Fig. 3.**
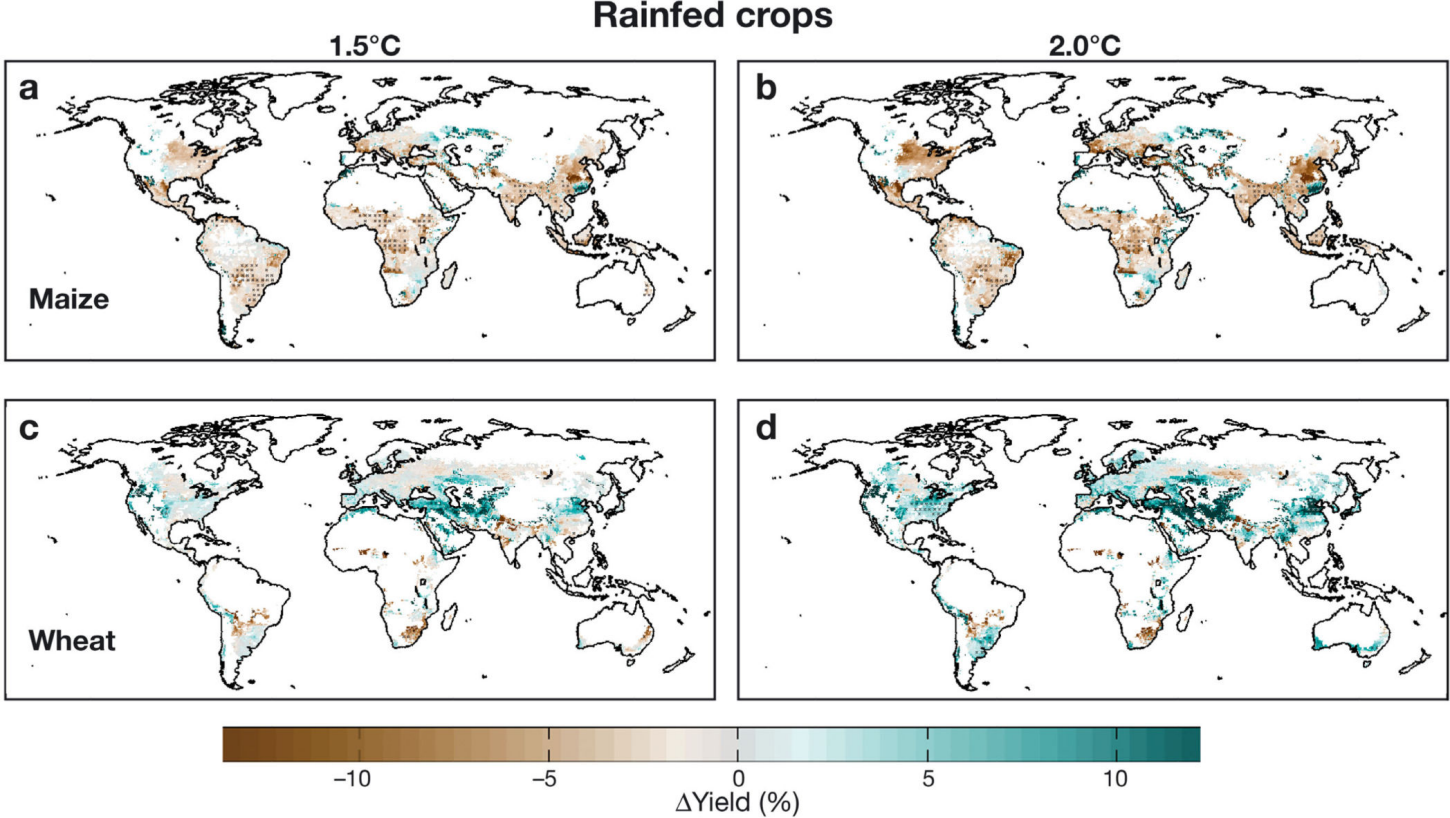

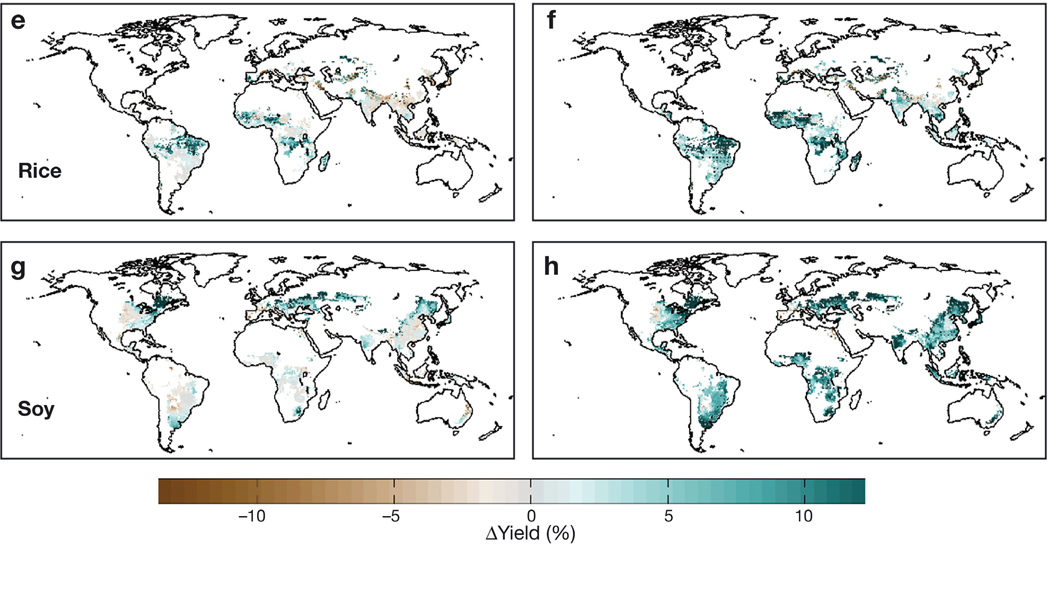
Median yield change projections for rainfed crops across 15 combinations of 5 HAPPI global climate models (GCMs) and 3 global gridded crop models (GGCMs). Hatch marks indicate regions where 70% of simulations agree on the direction of change. Projections include CO_2_ benefits at 423 and 487 ppm, respectively, for the +1.5° and +2.0°C Worlds

Fig. 3 presents median rainfed yield changes (across 15 GGCM/GCM combinations) for rainfed maize, wheat, rice, and soy under the +1.5° and +2.0°C Worlds in comparison to the current (~2010) climate (Rosenzweig et al. 2018 presented all model combinations for rainfed wheat). These median losses obscure substantial uncertainty between GGCMs (particularly related to the impacts of CO_2_) and among HAPPI GCMs (owing to variation in local temperature rise and precipitation changes); nevertheless, several patterns emerge.

Rainfed maize yields decline in most areas under the +1.5°C World (Fig. 3a). Rainfed wheat yield changes for the +1.5°C World are small (<5%) in major wheat belts of the North American Great Plains and Europe. Larger losses are evident in the northern Murray-Darling Basin of Australia, eastern South Africa, and northern Argentina, while western Asia and the North China Plain see substantial yield increases (Fig. 3c). +1.5°C World rainfed rice yield changes are also quite muted over the major production regions in Asia, while projecting increases over tropical Africa and South America (Fig. 3e). Rainfed soy projections improve yields over much of Eastern Europe and northwestern Asia in the +1.5°C World, also showing slight yield decreases over the interior of North America and equatorward portions of South America and East Asia, while gradually increasing toward the eastern US and poleward portions of South America and East Asia (Fig. 3g).

In the +2.0°C World, yields for the C3 crops (wheat, rice, and soy) improve in nearly all regions as CO_2_ effects largely overcome temperature challenges (Fig. 3d,f,h) (Asseng et al. 2015). Water-stressed regions show the largest gains, likely owing to the beneficial effects of elevated CO_2_ reducing transpiration losses (Deryng et al. 2016). As a legume, soy is not constrained by nitrogen limitations and thus responds strongly to rising CO_2_ (Kimball 2016). The C4 maize yields do not capture nearly the same level of CO_2_ benefit, with yields declining further as temperatures rise to the +2.0°C World (Fig. 3b).

Irrigated crops (Fig. S1 in the Supplement) respond in much the same way as rainfed crops, although they are largely immune to precipitation changes and do not benefit as much from the water retention benefits of CO_2_ given that water stress is controlled through farm management (photosynthetic stimulation still benefits C3 crops, but C4 crops are aided to a lesser extent). This leads to large irrigated maize losses over much of North America, China, and southern Europe, while yields are reduced for the irrigated wheat basket of South Asia under both the +1.5° and +2.0°C Worlds.

### 3.3. Uncertainty in agricultural production change projections

**Fig. 4.**
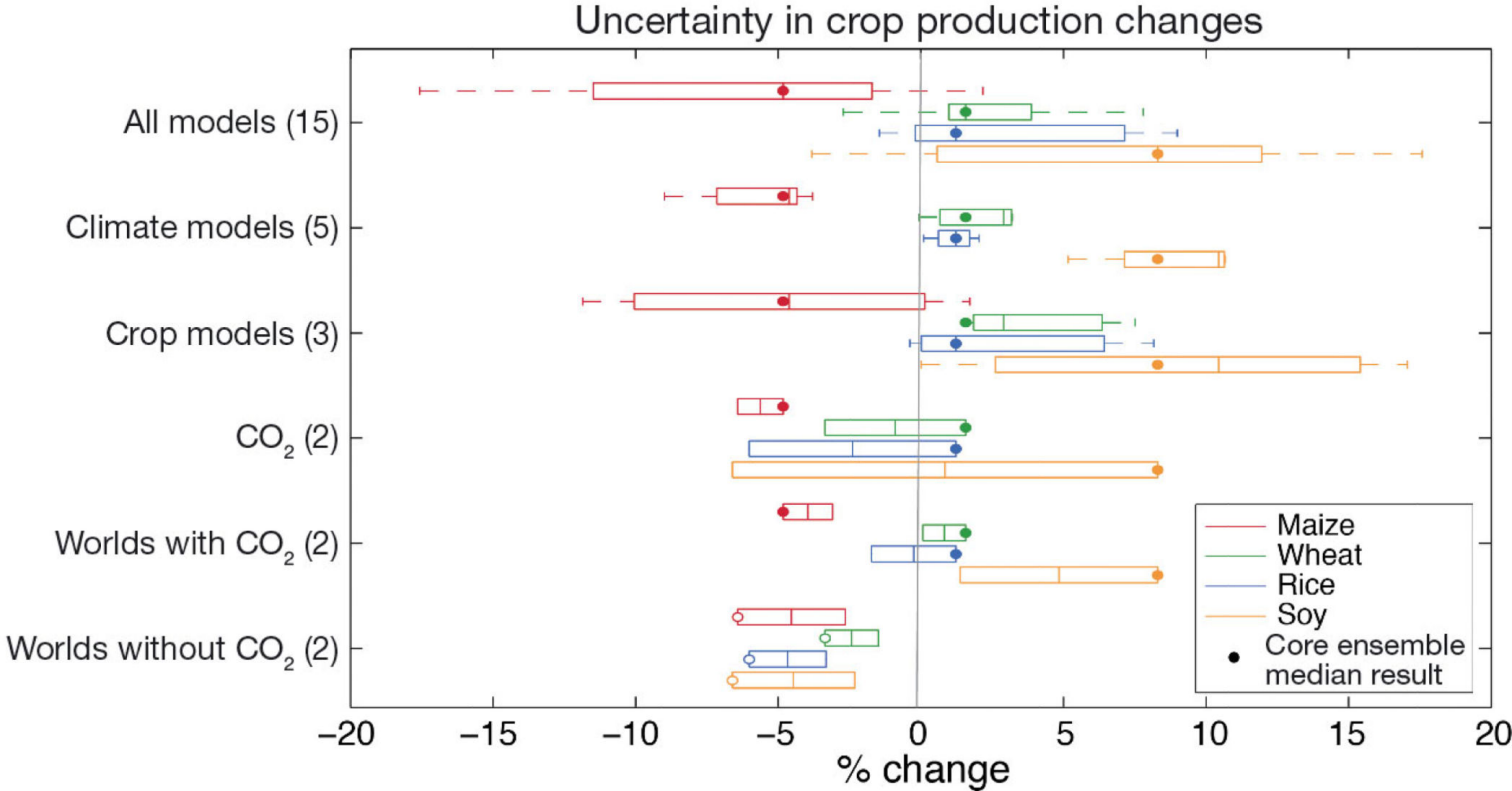
Uncertainty in global production change projections for the +2.0°C World for maize, wheat, rice, and soy owing to global climate models (GCMs) and global gridded crop models (GGCMs) with CO_2_ effects simulated. Dots indicate median production change from the core ensemble of all 15 GCM×GGCM combinations for each crop. For example, the climate models row shows the median of the 3 GGCMs for each of the 5 HAPPI GCMs, allowing an isolation of uncertainty from the climate model dimension. The effect of simulating CO_2_ effects is presented by comparing the median of all GCM×GGCM combinations with CO_2_ concentrations consistent with the +2.0 °C World (487 ppm) vs. the median of all GCM×GGCM combinations holding CO_2_ at current levels (390 ppm). For reference, the ‘Worlds’ rows present median changes in +1.5° and +2.0°C World production totals (across all GCM×GGCM combinations) both with and without the simulated effects of elevated CO_2_ (empty dots show the corresponding reference median of the +2.0°C World without CO_2_ effects). Production estimates generated by weighting yield changes by year 2005 crop areas (You et al. 2014). Box-and-whisker plots summarize each row’s ensemble (number of results listed in the *y*-axis label), including the median change (vertical line), interquartile range (IQR, edge of box), and whiskers extending to the last point within an additional 1.5 × IQR. Note that these production changes are the exogenous input for economic models, which may alter the distribution of agricultural areas endogenously in response to price and demand changes

Fig. 4 illustrates projections of global production change (compared to a future with no climate change) and major sources of uncertainty owing to climate and crop models as well as the inclusion of CO_2_ effects. These uncertainties (assessed here as the range in median responses across the full ensemble when 1 factor is isolated) are then compared to the differences between the +1.5° and +2.0°C Worlds. In the core scenario (+2.0°C World SSP1 with CO_2_ effects), there is strong agreement across the ensemble of all model combinations that maize production declines (median of −5%), wheat and rice production increases slightly (median of +1 to +2%), and soybean increases more substantially (median of +8%). Projection ranges determined by climate models are less than half of the range owing to the selection of crop models, and much of the crop model difference is related to the comparable uncertainty from CO_2_ benefits.

The extent to which elevated CO_2_ benefits crops remains an area of considerable ongoing debate within the literature (Long et al. 2006, Tubiello et al. 2007a,b, Ainsworth et al. 2008, Boote et al. 2010, Porter et al. 2014, O’Leary et al. 2015, Kimball 2016). Overall there is strong agreement that C3 crops (including wheat, rice, and soy) have a larger photosynthetic benefit than C4 crops (including maize), although both C3 and C4 species experience higher water use efficiency under elevated CO_2_ concentrations (Bongaarts 1994). Uncertainty in agricultural CO_2_ response stems largely from a lack of field experimentation for CO_2_ response, as existing data insufficiently sample the broad range of crop species, cultivar genetics, field environments, and management practices within the global agricultural sector (Leakey et al. 2012). Crop models have long been used to project climate change impacts including CO_2_ effects, as they combine response curves calibrated from available experimental data with a broader range of biophysical processes and plant–environment interactions represented in the model (Rosenzweig & Parry 1994, Asseng et al. 2013). Crop models can also simulate regional differences in CO_2_ response (Deryng et al. 2016) and gauge differential CO_2_ responses under extreme conditions (Durand et al. 2017). Reich et al. (2018) recently suggested that behaviors of C3 and C4 grassland plants may shift over time, although this effect is difficult to separate from inter-species competition and soil ecology.

CO_2_ benefits are widely expected to be non-negligible and positive (particularly for C3 crops), and thus it is not surprising that simulations without CO_2_ benefits (holding CO_2_ concentrations constant at 2010 levels) form the lower production extreme in the CO_2_ row of Fig. 4. Without CO_2_ benefits, projections for each crop show a decline in median production in comparison to a future without climate change, with soybean (a legume) responding most strongly given that it is rarely limited by soil nitrogen. The positive effects of CO_2_ also saturate at high concentrations, so these first increases of 33 and 97 ppm (for the +1.5° and +2.0°C Worlds) have a more potent benefit than would the next similar increases in a higher emissions pathway.

Differences between simulations with and without CO_2_ also illustrate the large global influence of CO_2_ effects compared to temperature and precipitation changes in the +2.0°C World. On a global production basis, the effects of regional precipitation increases or decreases largely cancel out (which helps reduce the GCM uncertainties), while warming and CO_2_ increases are more universal (see also agricultural region breakdown in Ruane et al. 2018). Schleussner et al. (2018) further found that higher CO_2_ levels only slightly decrease crop responses to temperature but shift the types of extreme events that regional agricultural systems respond to in the +2.0°C World (likely owing to water retention benefits aided by higher CO_2_ concentrations).

The magnitude of global crop production changes is generally exacerbated in the +2.0°C stabilization compared to the +1.5°C World, with rice changes shifting in direction (−2% in the +1.5°C World and +2% in the +2.0°C World; Fig. 4). Rosenzweig et al. (2018) showed that CO_2_ responses are a major basis for the simulated C3 crop production gains of the +2.0°C World scenario compared to the +1.5°C World, and also identified substantial uncertainty across specific GGCMs. The C4 maize crop sees an additional 2% decline moving from the +1.5° to the +2.0°C World. Without CO_2_ effects, temperature and precipitation changes cause the +2.0°C World to have lower production than the +1.5°C World for all crops.

## 4. GLOBAL MARKET RESPONSES

We explored the global economic effects of climate changes in these future worlds by employing the International Model for Policy Analysis of Agricultural Commodities and Trade (IMPACT) partial equilibrium model (Robinson et al. 2015) and the Future Agricultural Resources Model (FARM) computable general equilibrium model (Sands et al. 2014). IMPACT and FARM model outputs contributed to several efforts of the AgMIP Global Economic Modeling Team to analyze climate impacts on future agricultural markets, allowing their results to be placed in the context of the broader ensemble of AgMIP global economic models (Nelson et al. 2014a, Wiebe et al. 2015). Computable general equilibrium models simulate multiple sectors and generally have more capacity for other sectors to cover climate-induced losses in the agricultural sector, while partial equilibrium models simulate only the agricultural sector at higher complexity (Nelson et al. 2014b).

### 4.1. Representing +1.5° and +2.0°C World global agricultural markets

Climate shifts associated with the +1.5° and +2.0°C Worlds act as shocks on global agricultural production compared to a counterfactual future without climate changes. These shocks reverberate throughout a complex international agricultural system that is also affected by consumer demand for agricultural products, technological advances, socioeconomic change, and shifting policy priorities. These in turn transform the context of agricultural systems, prices, land use, and trade. Economic simulations test these trajectories through shared socioeconomic pathways (SSPs; O’Neill et al. 2017a), with specific conditions (e.g. population, GDP, land use restrictions, energy and food consumption) set according to the projection’s time horizon. Given difficulties in assessing market conditions more than several decades in the future, here we examine the impacts of a +1.5° or +2.0°C World assuming climate has stabilized in the 2050s. Despite HAPPI +1.5° and +2.0°C World simulations being pegged to 2106–2115, the biophysical shocks are consistent with the same climate occurring in 2050. This time horizon is similar to +1.5° and +2.0°C crossing points in many CMIP5 transient simulations, and is comparable to RCP4.5 and RCP6.0 climate conditions even as those scenarios continue toward much higher global warming later in the century and beyond (Collins et al. 2013, Ruane et al. 2018).

The core CGRA application examines the ‘Green Growth’ SSP1, wherein the world moves toward a more sustainable path with lower population growth, international cooperation, and technological development facilitating more efficient use of resources and stronger protection for the environment (O’Neill et al. 2017a, van Vuuren et al. 2017). Both global economic models simulated a counterfactual future in which the SSP1 pathway proceeds without climate impacts on agricultural production or additional mitigation efforts. These are compared to the same future pathway with agricultural production shocks determined by 3 GGCMI crop models each driven by 5 HAPPI GCMs, resulting in 15 future scenarios for global and regional assessment illustrating the additional burdens introduced by climate change on top of broader challenges of providing sufficient healthy food for a growing and developing population (FAO 2016). To understand the ramifications of societal development pathways, global economic models also simulated the ‘Middle-of-the-road’ SSP2 wherein current trends largely continue, resulting in higher populations and incomes, lingering trade barriers, income inequality, increased consumption of food and energy, and continued environmental degradation (Fricko et al. 2017, O’Neill et al. 2017a). The continuation of current dietary patterns and trends, in particular, places a growing strain on future SSP2 food systems and their global footprint.

The agricultural sector also has a mandate to play a role in global mitigation efforts given its substantial greenhouse gas emissions and historic land-use changes (Wollenberg et al. 2016). We therefore simulated example mitigation scenarios with the FARM model to explore how key policy incentives would affect agricultural markets. The FARM mitigation scenario uses CO_2_ prices applied to greenhouse gas emitters (including agricultural producers) and is constrained to emit ≤800 Gt CO_2_ globally from 2011 through 2050. CO_2_ emissions start at 32.9 Gt CO_2_ in 2011 and decline to 7.1 Gt CO_2_ in 2050. This is consistent with an emissions pathway with a cumulative emissions limit of 1000 Gt CO_2_ from 2011 through 2100 (consistent with a +2.0°C stabilization). The FARM model solves for global CO_2_ prices at each time step to meet an exogenous global emissions target.

GGCM yield outputs (including CO_2_ effects) were processed within the CGRA framework to meet the input requirements of the global agricultural economics models. Aggregation of GGCMI yield change ratios to countries and regions used 2005 agricultural area information from the Spatial Production Allocation Model database for area-weighting and total production calculations (SPAM; You et al. 2014). To inform the many agricultural commodities simulated by the economic models, climate impacts on crops not explicitly modeled by GGCMI were estimated on a country level using a combination of species similarity (e.g. C3 vs. C4; legumes), experimental literature, and constraints to prevent spurious production changes beyond ±25%. Future agricultural production includes the effects of improved farm technologies and yield gap closures associated with socioeconomic development in each SSP; however, these effects are included in all simulations (including the no-climate-change counterfactual) so that we can gauge the specific effects of climate shocks and mitigation. Global economic simulations were also conducted driven by GGCM results that exclude CO_2_ effects in order to understand the market effects of this major biophyscial uncertainty.

### 4.2. Agricultural market change projections

**Fig. 5.**
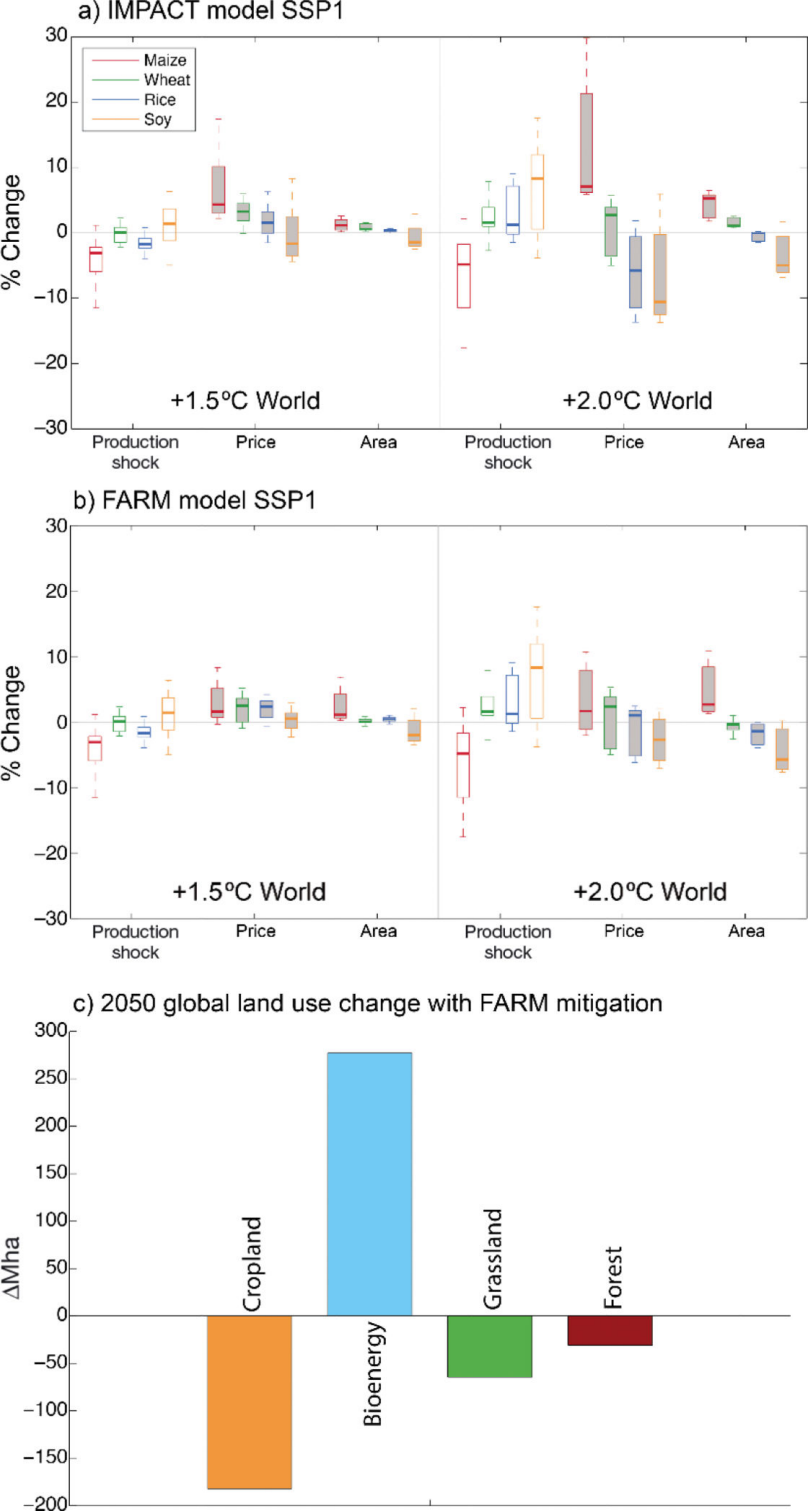
Summary of global economic mo del simulations under +1.5° and +2.0°C Worlds for the (a) IMPACT model and (b,c) FARM model. Panels (a) and (b) show production changes (from global gridded crop models, GGCMs) as well as area and price shifts (from economic models) for major cereals under a nomitigation scenario (shared socioeconomic pathway 1, SSP1) with direct climate impacts on global production including CO_2_ effects (15 combinations from 3 GGCMs and 5 global climate models, GCMs). (c) Area changes for major land use types associated with bioenergy focused mitigation scenarios for the +2.0°C World. Box-and-whisker plots as described in Fig. 4

Fig. 5 summarizes agricultural market responses to direct climate impacts associated with a +1.5° or +2.0°C World compared to a future without climate change. Fig. 5a,b shows how production shocks on existing croplands (with CO_2_ effects as described in Section 3) affect prices, which in turn drives expansions or reductions in cultivated areas motivated by profit and yield potentials. The overall relationship between production shocks, prices, and cultivated area is complicated by dependence on the geographic pattern of yield increases and decreases, the availability of agricultural lands, costs associated with transitions in farm systems and trading partners, and the possible substitution of one crop for another (e.g. livestock may consume wheat-based feed if maize becomes more expensive).

In the +1.5°C World, reductions in maize and rice production drive up their prices, increasing area to make up for production gaps. Wheat prices and area also increase despite nearly flat global production levels, likely carried upward by pressure on maize and rice. Increases in soy production lead to declining area and prices that are somewhat lower in IMPACT but relatively flat in FARM. Maize production declines further in the +2.0°C World; however, production for wheat, rice, and soy increase compared to a future without climate change (owing largely to uncertain CO_2_ effects on C3 crops). This results in continued upward pressure on maize prices and area but an increasing number of simulations showing declines in prices and area for wheat, rice, and soy.

Fig. 5c breaks down the additional pressure on agricultural land use in response to ambitious mitigation targets that could play a role in achieving a +2.0°C climate stabilization. FARM simulation of the +2.0°C mitigation pathway (without any direct effects of climate change on crop production) indicates disruption to global land use as mitigation policies are implemented as bioenergy crops expand to 284 Mha in 2050 to provide a green energy source on a scale that helps achieves the +2.0°C World (bioenergy accounts for only 7.1 Mha in the non-mitigation SSP1 reference). Land devoted to bioenergy comes largely from croplands (−16% of reference areas) and grasslands (−2% of reference areas), which would require substantial intensification in remaining agricultural systems to meet food demands. A related intercomparison of global economic models also found substantial decreases in land devoted to food production in response to mitigation policies (van Meijl et al. 2018).

### 4.3. Uncertainty in global agricultural market projections

**Fig. 6.**
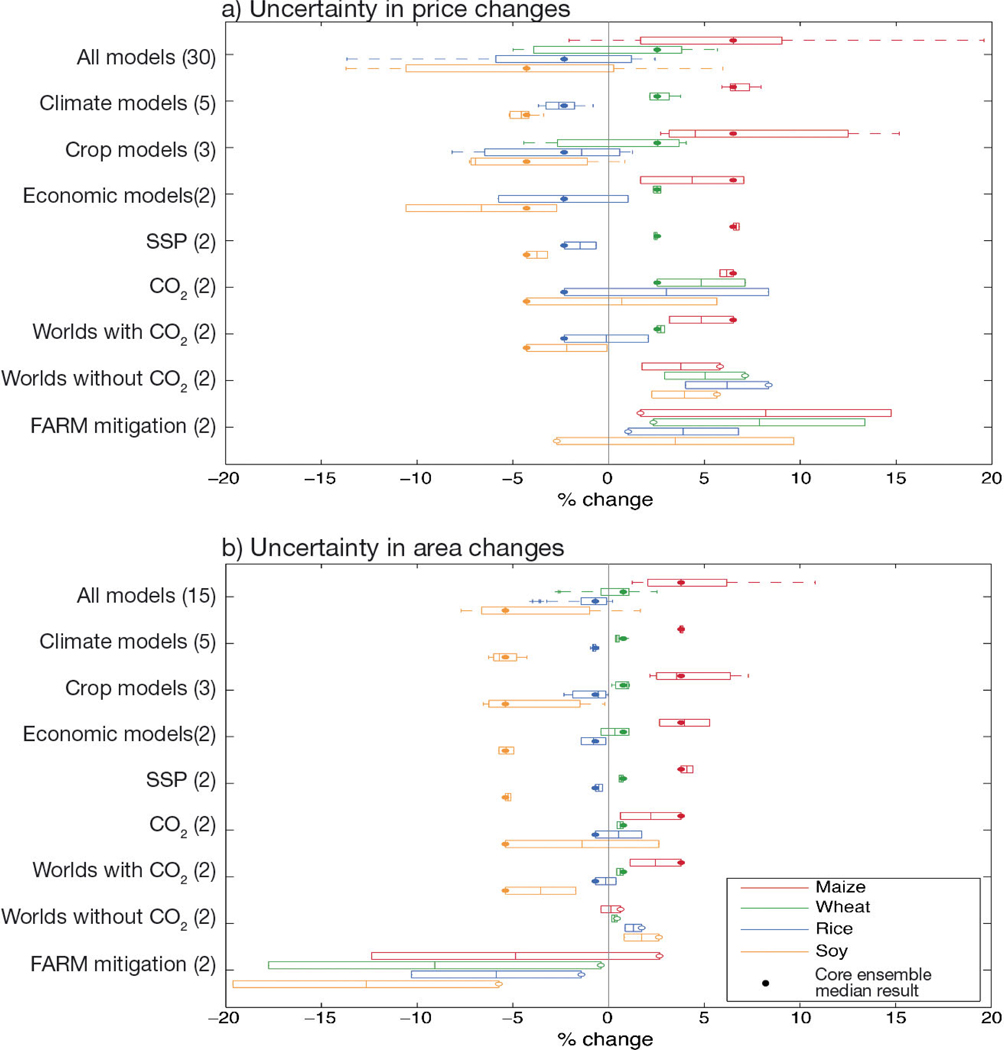
Uncertainty in (a) global prices and (b) global cultivated area for maize, wheat, rice, and soy in the +2.0°C World with CO_2_ effects, shared socioeconomic pathway 1 (SSP1), and no mitigation. Rows 2–4 indicate uncertainty in isolated dimensions expressed as the range in the median of the other dimensions of the core model ensemble (total of 5 global climate models [GCMs] × 3 global gridded crop models [GGCMs] × 2 economic models). The ‘CO_2_’ row shows differences between median crop production estimates in the +2.0°C World with and without CO_2_ impacts; ‘SSP’ shows differences between medians of SSP1 and SSP2; ‘Worlds’ show the median price and area changes of the +1.5° and +2.0°C Worlds with and without the effects of CO_2_; ‘FARM Mitigation’ shows differences between median simulations with direct climate impacts only and those that also include the carbon price-based mitigation scenario. Filled dots show core ensemble medians for each crop, while empty dots in the last 2 rows represent the reference +2°C World without CO_2_ and the +2.0°C World from the FARM model, respectively. Box-and-whisker plots as described in Fig. 4

Fig. 6 displays global crop price and crop area projections for a core scenario featuring the SSP1 +2.0°C World including CO_2_ effects and no additional mitigation. It further explores major sources of uncertainty from 3 types of models (climate, crops, and economics) as well as deviations from this core scenario driven by the inclusion of CO_2_ effects, SSP, and a specific mitigation scenario applied to the FARM economic model. Uncertainty from various factors (assessed here as the range in median responses across the full ensemble when 1 factor is isolated) are compared to differences between the +1.5° and +2.0°C Worlds to place model and scenario uncertainty in the context of the decision space targeted by the Paris Agreement. The full model ensemble features 30 combinations (5 GCMs × 3 GGCMs × 2 global economic models) with considerable uncertainty, although the ensemble strongly indicates increases in the price and area of maize and wheat while rice and soy see price and area declines.

Climate models are not a major source of price uncertainty and have very little influence on crop areas owing to the aggregating effects of global production and market forces. Crop models drive substantial price and area uncertainty for all crops. Crop model uncertainty is largely comparable to uncertainties from the inclusion of CO_2_ effects for C3 crops (wheat, rice, and soy), with LPJmL tending to have larger CO_2_ effects than the other models. Maize (a C4 crop with lower responses to CO_2_) sees additional crop model uncertainty likely owing to a stronger thermal response within pDSSAT. Overall differences in price and area changes across the 4 cereal crops indicate a need to include direct simulation of more commodities for future market assessments.

Relative to the IMPACT model, in the FARM model production shocks lead to slightly smaller price changes, but larger area changes for these 4 primary cereal crops (see Fig. 5). This is likely due in part to IMPACT only directly simulating the agricultural sector, but including a wider number of competing crop types, while the FARM model simulates a wider variety of competing land uses and buffers prices through responses in other sectors. IMPACT and FARM also differ in assumptions on land expansion, agricultural productivity growth, demand, and the possibilities for substitution between commodities (Nelson et al. 2014b); the latter of which likely explains why wheat prices are more comparable between economic models than the other commodities. Although raw prices and land use have large differences between SSP1 and SSP2, their proportional response to production shocks is relatively unaffected by SSP selection.

Key emergent messages are apparent in the projections, even as median differences in the full ensemble between the +1.5° and +2.0°C Worlds are on the same order as (and often smaller than) uncertainties in crop and economic models. When CO_2_ effects are included, median increases in maize and wheat prices and area exist for both Worlds, as do decreases in soy price and area. The direction of change for rice prices and area shifts from increases in the +1.5°C World to decreases in the +2.0°C World.

Uncertainty from the inclusion of CO_2_ benefits is particularly important given that simulations of the +2.0°C World without CO_2_ benefits reverse all price and area decreases, resulting in clear pressure for higher prices and expanded cropping area for all commodities relative to a world without climate change. When CO_2_ is included, the 2.0°C World has lower prices than the 1.5°C World for C3 crops and reduced areas for rice and soy (wheat goes up slightly due to substitution effects), but without CO_2_ benefits, the +2.0°C World has higher prices and areas for all crops due to warming and rainfall changes. As such, the considerable uncertainty in CO_2_ effects assuredly propagates into the global economic outlook, although the range between simulations with and without CO_2_ effects is likely higher than the true CO_2_ uncertainty. Previous studies (e.g. Nelson et al. 2014a,b, Asseng et al. 2015, Wiebe et al. 2015) did not include CO_2_ effects; however, CO_2_ effects are widely understood to be positive even as the magnitude of this benefit is uncertain (Leakey et al. 2012, Kimball 2016). If CO_2_ effects are indeed overestimated in current crop models, this would indicate that the +1.5° and +2.0°C World projections are likely to reduce availability of convenient food substitutes, drive up crop prices, and heighten land resource competition.

The ‘FARM Mitigation’ row of Fig. 6 compares the no-mitigation and mitigation simulation ensemble within the FARM economic model, shining a spotlight on the ways in which the implementation of a mitigation strategy can cause substantial disruption as the agricultural sector seeks to play a role in emissions reduction. The dynamic carbon price in the FARM mitigation scenario is oriented to emitters, which dramatically increases energy costs in farm production as well as land use competition from bioenergy crops (Fig. 5c). As a result, a further 10–15% of area for the 4 cereal crops is reallocated and prices rise 5–10% above the no-mitigation scenario. These FARM mitigation scenario changes are larger than the direct impacts of climate change associated with the +1.5° and +2.0°C Worlds. FARM results represent only 1 example of a potential mitigation strategy, but a related intercomparison of global economic models also highlighted the benefit of harmonized economic model assessment, and agreed that the costs of mitigation to achieve +1.5° and +2.0°C Worlds may likely exceed the costs of adaptation to those new climate conditions (van Meijl et al. 2018). Mitigation costs also lead to a corresponding increase in hungry populations and food insecurity (Hasegawa et al. 2018) compared to the climate changes alone. As a contrast (not evaluated here), Springmann et al. (2017) noted that efforts to reduce food consumption (e.g. through the promotion of more sustainable diets) can lead to a reduction in demand that relieves a portion of the pressure on agricultural lands and emissions.

## 5. REGIONAL INTEGRATED ASSESSMENT OF GLOBAL MARKET PRESSURES AND LOCAL CLIMATE VULNERABILITY

Analysis at the global scale may overlook substantial local challenges and opportunities for farmers and other agricultural sector stakeholders, and too often gives the impression of homogeneous regional responses, despite extensive heterogeneity in households, environmental conditions, and farming systems within any given region. Here we apply elements of AgMIP’s regional integrated assessment (RIA) protocol to examine the +1.5° and +2.0°C Worlds from a regional perspective (Rosenzweig et al. 2017). Crop models were configured according to field experiments in the case study region, providing local cultivars and farm management that can be combined with regional soil profiles and weather conditions (in contrast to the more generic configurations used by GGCMs). We simulate future systems under the new climate stabilizations and farm management within representative agricultural pathways (RAPs) developed in conjunction with local stakeholders to reflect local agricultural development (Valdivia et al. 2015). This allows an analysis of economic outcomes for a survey of rural households in case study regions (Antle et al. 2015).

CGRA regional case studies examined biophysical impacts caused by local climate changes (including CO_2_ effects) within the +1.5° and +2.0°C Worlds, as well as the immediate and long-term effects of shifts in global commodity prices as mitigation policies are enacted and climate shifts impact other regions. Case studies are not intended to be comprehensive, but were selected along a southeast to northwest cross-section of US agricultural systems as examples of developed country impacts, with a developing country example drawn from Pakistan. Biophysical impacts were assessed at Camilla, Georgia (southeastern US), Ames, Iowa (US Midwest), and Greeley, Colorado (US Front Range) using the Decision Support System for Agrotechnology Transfer Cropping System Model (DSSAT-CSM; Hoogenboom et al. 2015). In contrast, the analysis of Pacific Northwest wheat systems utilized the Tradeoff Analysis Model for Multi-Dimensional Impact Assessment (TOA-MD; Antle et al. 2014) to evaluate the economic and environmental (greenhouse gas) performance of those systems adapted to low greenhouse gas emissions scenarios and an SSP1 storyline using a suite of model-based inputs that included results from the DeNitrification-DeComposition (DNDC) crop model (Gilhespy et al. 2014), mitigation policy incentives, and life cycle analysis. The TOA-MD model was also applied for cotton–wheat systems in 5 villages within Punjab, Pakistan, integrating DSSAT yield impacts, IMPACT price changes, and RAPs developed in collaboration with local experts and stakeholders (Ahmad et al. 2015). We summarize CGRA case studies briefly below, with more detailed analysis to be provided in forthcoming partner CGRA studies on Pakistan economics and the effects of mitigation on the US Pacific Northwest.

### 5.1. Representing local farm and market effects of +1.5° and +2.0°C Worlds

Commodity price changes (compared to a counterfactual future without climate change) for each case study region were supplied by IMPACT SSP1 simulations for all GCM/GGCM combinations, and these differ from global prices due to local supply, demand, and barriers to trade. Future farming systems in DSSAT and TOA-MD were represented by the sustainability-oriented ‘Green Road’ RAP that is associated with SSP1 (Valdivia et al. 2015). Biophysical impacts in case studies were driven by local climate scenarios differentiated from the global scenarios in that they (1) imposed HAPPI climate shifts upon local climate observations (supplied by the US Historical Climatology Network and the Pakistan Meteorological Department) rather than gridded climate data; and (2) adjusted daily climate series according to monthly shifts in mean conditions as well as changes in the number of rainy days and the distribution of daily maximum and minimum temperatures (Ruane et al. 2015a). An example of monthly scenario conditions in Pakistan is provided by Rosenzweig et al. (2018).

### 5.2. Local yield impact case studies for +1.5° and +2.0°C Worlds

**Fig. 7.**
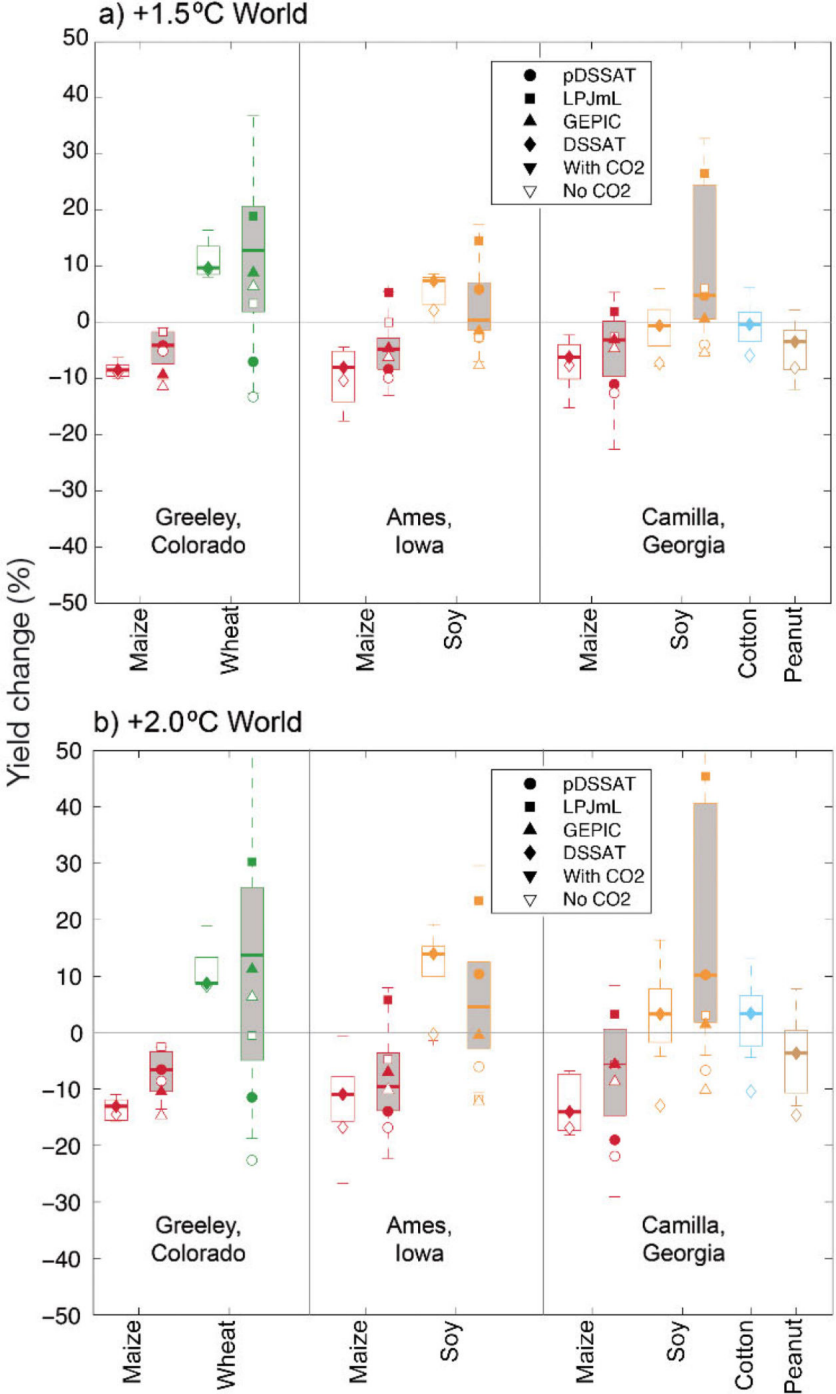
Overview of regional crop modeling results for case studies in the US for the (a) +1.5°C World and (b) +2.0°C World. Local DSSAT results (across 5 HAPPI global climate models, GCMs) presented as unfilled box-and-whisker plots, while filled box-and-whiskers show corresponding global gridded crop model (GGCM) results under the same irrigation scheme. Symbols mark the median change for each GGCM (across 5 HAPPI GCMs), with filled symbols including CO_2_ effects and unfilled symbols using constant CO_2_ (no simulated benefit from CO_2_). Note that DSSAT results are a blend of 3 rainfed and 3 irrigated treatments for Camilla, Georgia, while only rainfed GGCM results are presented

Fig. 7 presents yield impacts over the US case study cross-section from both the local and global crop modeling perspectives. Similar to the global signal, maize yields decline at all 3 locations, while soy yields mostly increase. Locally-calibrated DSSAT and global crop model projections overlap and agree on the sign of median yield changes for all but Camilla soy in the +1.5°C World (potentially due to multiple water management treatments in the DSSAT results). There is a notable increase in uncertainty for the GGCMs; however, by isolating the median changes from the 3 GGCMs, it is apparent that GGCM differences are driving this uncertainty (if GCMs were the cause, the GGCM median would cluster near the center of the distribution). As was apparent in the global production results (Section 3), differences between simulations with and without CO_2_ effects point to CO_2_ responses as a major contributor to inter-GGCM spread for C3 crops (particularly in the +2.0°C World). LPJmL, in particular, shows reduced losses and elevated gains for all case study crops compared to the other models, corresponding with larger CO_2_ responses. Median pDSSAT and local DSSAT results (which come from the same underlying process model) match very closely for the Ames site; however, differences at Camilla and Greeley likely stem from their use of different observational datasets and procedures for the configuration of cultivars and management. Local DSSAT application also provides additional information on peanuts and cotton at the Camilla site (these crops were not simulated by the GGCMs).

### 5.3. Regional impact assessment case studies for +1.5° and +2.0°C Worlds

Regional implications of the +1.5° and +2.0°C Worlds are driven by the balance of local yield changes and shifting market prices, as well as policy and development trends that may counteract or exacerbate impacts on farm returns. Urban populations and non-farmer rural households would not benefit from rising prices for farm output, but will experience the price impacts as well as disruptions in commodity supply chains. This may lead to situations where farmers benefit from higher market returns even as consumers struggle to cope with higher food prices, or vice versa.

**Fig. 8.**
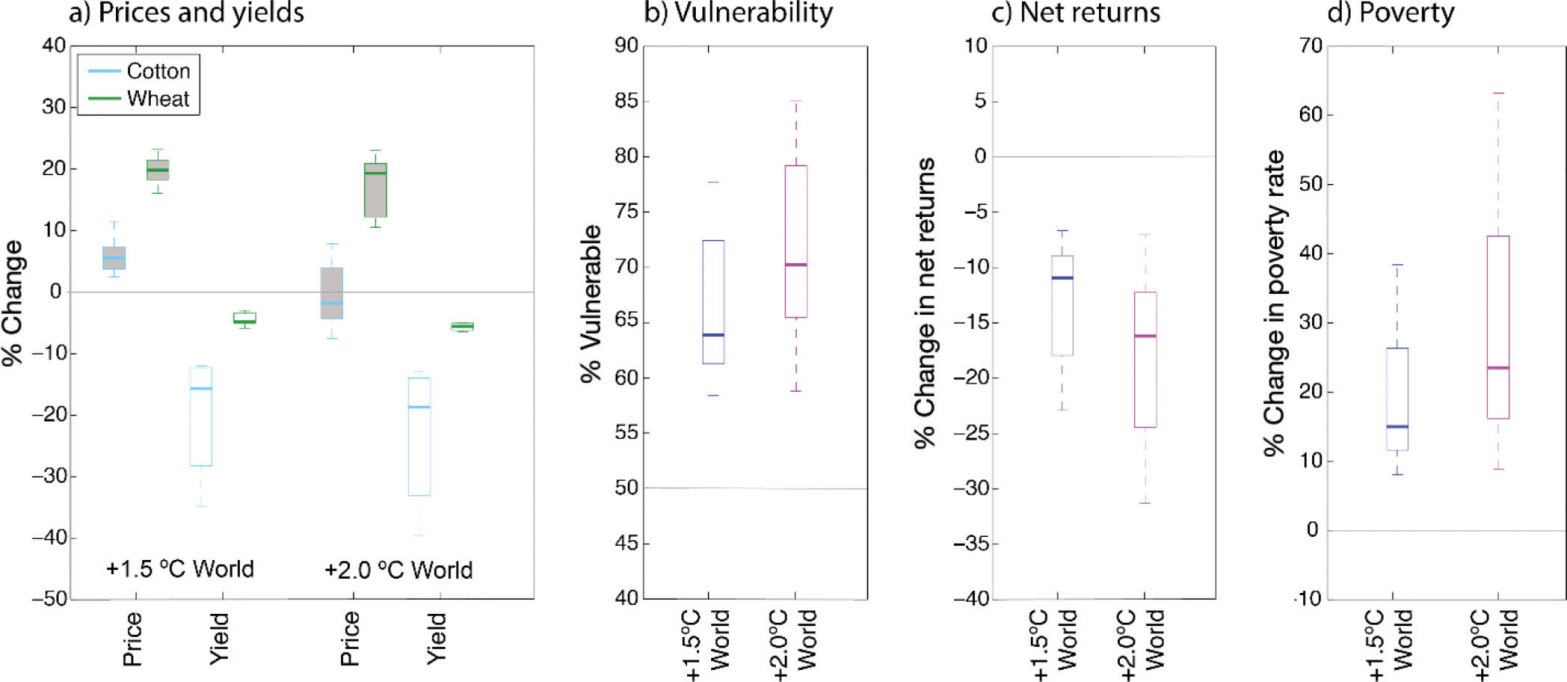
Summary of economic impacts for cotton–wheat systems in Punjab, Pakistan. (a) IMPACT shared socioeconomic pathway 1 (SSP1) no-mitigation Pakistani price and DSSAT yield changes for 2050 climate stabilizations that drive household economic simulations; (b) percentage of farm households that are vulnerable under both the +1.5° and +2.0°C World scenarios; (c) percentage change in net farm returns; (d) percentage change in poverty rate (per capita income <US$1.25 d^−1^; as compared to reference SSP1/RAP rate of 8.2% in 2050). Box-and-whisker plots (parameters as in Fig. 4) show household economic projections combining 15 IMPACT simulations with different GCM × GGCM combinations combined with corresponding DSSAT yield changes from 5 GCMs

In cotton–wheat systems in Punjab, Pakistan (Fig. 8), irrigated cotton yields show strong sensitivity to temperature increases that overwhelms any positive CO_2_ benefit, with median yield declines in both the +1.5° and +2.0°C Worlds (14 and 19% losses, respectively; Fig. 8a). Wheat yields also decline, but at a lesser rate (5 and 6% losses, respectively). Farmers facing falling yields see some relief in wheat prices that rise ~20% in the 2050 IMPACT SSP1 no-mitigation simulation, and these are even higher than the global prices due to demand and trade networks within South Asia. Cotton price changes are positive (+5%) in the +1.5°C World but then turn negative (−2%) in the +2.0°C World. This turn reflects higher yields in other cotton production regions, which respond strongly to higher CO_2_ and are further from critical temperature thresholds that challenge Punjab cotton in the +2.0°C World (recall cotton projections for Camilla, Georgia; Fig. 7).

Results from the TOA-MD model help us understand ramifications of global price changes and regional crop yield impacts on Punjabi cotton–wheat systems (Fig. 8b–d). The percentage of vulnerable households (Fig. 8b) indicates the proportion of households that are at risk of losing income due to the conditions imposed by the +1.5° and +2.0°C scenarios. A median of 64% of households are vulnerable in the +1.5°C World, driven by yield declines in cotton (the critical cash crop) that outpace price increases and lead to a decrease in net farm returns (−11%; Fig. 8c). In the +2.0°C World, household vulnerability rises to 70% and net farm returns decline further (−16%) as cotton yield declines further while cotton price impacts turn negative. The percentage of vulnerable households does not reach 100% as some farmers benefit from the price increase, but the climate impact scenarios raise poverty rates (per capita income less than US$1.25 d^−1^) by a median of 14 and 24% in the +1.5° and +2.0°C Worlds, respectively. Regional economic outputs (Fig. 8b–d) do not benefit from the spatial and market aggregations in global economic assessments, resulting in substantial regional uncertainty from local climate projections manifested in crop yield projections in addition to smaller effects from the suite of global price projections. The Pakistani case study thus offers the perspective of a region facing acute impacts on a key cash crop, underscoring the need to consider regional impacts even as global impacts may appear more manageable.

The analysis of Pacific Northwest dryland wheat systems in the US conducted by Antle et al. (2018) provides an important additional perspective of policy makers weighing incentives for farmer adoption of mitigation options such as those that could help achieve +1.5° or +2.0° C Worlds. Their assessments using the TOA-MD model addressed 3 key factors facing farmers on a 2030 time horizon: (1) changes in crop prices and costs of production associated with low-emissions scenarios; (2) policy incentives and technology adoption for emissions reductions through soil carbon sequestration; and (3) policy incentives and technology adoption for production of biofuels in a camelina *Camelina sativa*/ wheat rotation. Due to the focus on adaptation of these systems in the near term, relatively small changes in crop productivity due to climate change and CO_2_ fertilizer were found. A sensitivity analysis to crop prices, costs of production, carbon prices, and biofuel prices was also conducted to determine example policy incentives that would attract farmer participation. Results indicated that 40% of farmers would participate, given that policy incentives approximately doubled farm incomes when adopting low-greenhouse gas emitting systems (aided by somewhat higher crop prices). More aggressive policy incentives (carbon prices of $75 t^−1^ of C; high biofuel crop subsidies) would increase adoption to 70% and triple farm incomes. These interventions would in turn reduce the net global warming potential of emissions of these systems by 20 to 35% (see Antle et al. 2018 for full details). The Pacific Northwest case study thus demonstrates that mitigation policies can be quite beneficial to farmers if incentivized by policymakers, although the latter must find the resources to support these incentives.

## 6. DISCUSSION

AgMIP’s CGRAs of the agricultural implications of +1.5° and +2.0°C warming provide insights into future challenges and opportunities for mitigation and adaptation. This first CGRA application illustrates the potential of linked models, scenarios, and case studies to provide consistent and multi-perspective insight for stakeholders in the agricultural sector and beyond. Assessment of the +1.5° and +2.0 °C Worlds also identified key sources of uncertainty and opportunities to improve the multi-discipline, multi-scale, and multi-model analysis framework of the CGRA.

### 6.1. Summary of findings

Agriculture in the +1.5° and +2.0°C Worlds is characterized by differential impacts across regions and farming systems. This finding of differential outcomes is also projected for other sectors at relatively low levels of global warming (O’Neill et al. 2017b). Yields for C3 crops (wheat, rice, soy) are higher in the +2.0°C World than the +1.5°C World, while C4 maize yields decline further (particularly in the tropics). Temperature, precipitation, and yield changes can be acute for specific regional farming systems, but on aggregate, the detrimental effects of increasing temperatures are offset to an extent by the beneficial impacts of elevated CO_2_ (particularly for C3 crops) and direct effects are smaller than those projected for RCP4.5, RCP6.0, and RCP8.5 at the end of the century (Rosenzweig et al. 2014). Without CO_2_ effects, yields for all 4 cereals decline at an increasing rate with global warming between the +1.5° and +2.0°C Worlds, which is an important caveat given continued uncertainty in CO_2_ response and its influence on all aspects of this CGRA assessment.

Projected production changes alter prices and increase land use and agricultural expansion pressures even as international trade and crop substitution effects buffer the deepest impacts. Global changes mask starker contrasts in outcomes at a regional scale, as yield changes often outpace price changes as was shown to negatively affect cotton-wheat systems in Pakistan. Yields on a cross-section of US sites show both positive and negative outcomes, but also highlight crop model uncertainty in field configuration and the extent of CO_2_ benefit. A hypothetical +2.0°C World mitigation scenario simulated by the FARM model would be quite disruptive in the agricultural sector, as dramatic expansion of bioenergy land use comes at the expense of croplands and grasslands, thereby raising crop prices beyond the impacts of direct climate impacts alone (an effect that would be even larger to meet the +1.5°C global constraint). In contrast, analysis of wheat systems in the northwestern US provides an example where farmers gain substantially from climate policies and price increases that incentivize carbon sequestration and biofuel production.

### 6.2. Priorities for future development

The Paris Agreement challenged society to limit global climate changes to a level that would minimize damages and be close enough to current conditions to facilitate practical adaptations. These targeted climate stabilizations therefore feature climate changes that are quite small compared to the higher RCPs and end-of-century conditions examined in previous assessments, leaving direct impact uncertainties among models (climate, crop, and economics) that are comparable in many cases to the magnitude of overall projected changes and the difference between stabilization Worlds (recall Figs. 4 & 6).

Field experiments of fundamental biophysical responses and global datasets of agricultural management continue to be bottlenecks holding back model development (Jones et al. 2017, Porter et al. 2017). Improvement of the CO_2_ response is particularly critical given that this uncertainty has the potential to shift the sign of global production changes with far-reaching repercussions. Global and regional economic impacts are likely sensitive to the time horizon of climate stabilization, which was set at 2050 here, but could be explored in different years given uncertainty in climate sensitivity and emissions policy (Rosenzweig et al. 2018, Ruane et al. 2018). Future CGRA applications would also benefit from more direct coupling of models to examine feedback loops, the establishment of commodity-based modeling networks (e.g. Asseng et al. 2015) and regional communities of modelers (e.g. Kollas et al. 2015), and the configuration of additional regional integrated assessments linking climate, crops, economics, and stakeholders examining regional vulnerability and options for adaptation and mitigation (such as was used in Pakistan and the US Pacific Northwest).

The CGRA framework could also be used in collaboration with the broader integrated assessment modeling community to evaluate the food–energy–water nexus under specific future pathways defined by SSPs, RAPs, and policy trajectories (Ruane et al. 2017). These could include the Paris Agreement’s nationally determined commitments or policies oriented toward achieving the Sustainable Development Goals (UN 2015). CGRA evaluation of mitigation strategies on the global (IMPACT and FARM) and regional (Pacific Northwest incentives) levels demonstrate the importance of continued identification and evaluation of a broad portfolio of mitigation strategies (and the need to facilitate consistent multi-model mitigation assessments). These include mitigation oriented toward both production and consumption, for example the climate-smart intensification of current agricultural lands, alternative dietary pathways, land-use restrictions, and approaches for bioenergy with carbon capture and storage (BECCS) and associated policy incentives. These mitigation options must also consider the perspective of farmers, agricultural stakeholders, and policymakers in countries where agriculture remains a major portion of gross domestic product and those regions with high land and water resource competition.

## Supplementary Material

Supplement

## Figures and Tables

**Table 1.** Overview of models used in Coordinated Global and Regional Assessments (CGRA) +1.5° and +2.0°C World frameworks. CGRA processed global climate model outputs provided by HAPPI, and used them to generate agricultural model input scenarios for global and local crop models

Row no.	Model (key reference)	Scale	Discipline	Inputs from	Outputs go to rows	Notes

1	CanAM4 (von Salzen et al. 2013)	Global + Local	Climate	HAPPI	6–9	Climate conditions provided as monthly statistics from multi-member global ensemble, aggregated to seasonal changes for Global Gridded Crop Model Intercomparison (GGCMI) applications (rows 6–8) or combined with local weather observations for local crop model applications (row 9). Simulated 2010 conditions and scenarios for +1.5° and +2.0°C Worlds.
2	CAM4-2degrees (Neale et al. 2013)	Global + Local	Climate	HAPPI	6–9	
3	HadAM3P (Massey et al. 2015)	Global + Local	Climate	HAPPI	6–9	
4	MIROC5 (Shiogama et al. 2014)	Global + Local	Climate	HAPPI	6–9	
5	NorESM1 (Iversen et al. 2013)	Global + Local	Climate	HAPPI	6–9	
6	pDSSAT (Elliott et al. 2014)	Global	Crops (site-based process model)	1–5	11–12	Global gridded version of DSSAT. Future yields linearly interpolated between sensitivity test conditions. Run with and without CO_2_ effects.
7	LPJmL (von Bloh et al. 2018)	Global	Crops (ecosystem model)	1–5	11–12	Future yields linearly interpolated between sensitivity test conditions. Run with and without CO_2_ effects.
8	GEPIC (Folberth et al. 2012)	Global	Crops (site-based process model)	1–5	11–12	Global gridded version of EPIC. Future yields emulated according to quadratic parameters fit to sensitivity test outputs. Run with and without CO_2_ effects.
9	DSSAT (Hoogenboom et al. 2015)	Local	Crops	1–5	13	Incorporates representative agricultural pathway (RAP) to represent future system management. Run with and without CO_2_ effects.
10	DNDC (Gilhespy et al. 2014)	Local	Crops	—	13	Examines direct climate impacts on 2030 time horizon and emissions from current and low-emissions management.
11	IMPACT (Robinson et al. 2015)	Global	Economics	6–8	13	Utilizes SSP1 with no mitigation, comparing future with climate impacts on agriculture to counterfactual future without climate impacts. Also simulated SSP2
12	FARM Sands et al. 2014)	Global	Economics	6–8	6–8	and a mitigation scenario based on carbon prices and land-use restrictions. FARM also examined bioenergy-focused mitigation scenario for reference.
13	TOA-MD (Antle et al. 2014)	Regional	Economics	9–11	—	Incorporates RAP to represent future agricultural systems, socioeconomic conditions, markets, and policies.
