# Supplementary material for: Biophysical and economic implications for agriculture of +1.5° and +2.0°C global warming using AgMIP Coordinated Global and Regional Assessments
Source: Clim Res. Author manuscript; Available in PMC 2020 Nov 4. (PMC7641099; doi:10.3354/cr01520)
Supplement: Supplement [file NIHMS1529641-supplement-Supplement.pdf]

The following supplement accompanies the article

Biophysical and economic implications for agriculture of +1.5° and +2.0°C global warming using AgMIP Coordinated Global and Regional Assessments

Alex C. Ruane*, John Antle, Joshua Elliott, Christian Folberth, Gerrit Hoogenboom, Daniel Mason-D'Croz, Christoph Müller, Cheryl Porter, Meridel M. Phillips, Rubi M. Raymundo, Ronald Sands, Roberto O. Valdivia, Jeffrey W. White, Keith Wiebe, Cynthia Rosenzweig

*Corresponding author: alexander.c.ruane@nasa.gov

Climate Research 76: 17–39 (2018)

S1. GGCMI Yield emulation.

GGCMI Phase 2 requested 756 unique combinations of imposed CO_2, temperature, water, and nitrogen changes under the no-adaptation case used in this study, with each simulating the 1980-2009 (30-year) period across the entire globe for maize, wheat, rice, and soy (**Table S1**).

Table S1: GGCMI sensitivity tests for carbon dioxide [CO_2], temperature change (ΔT), precipitation change (or change in water; ΔW), and nitrogen fertilizer (N). Conditions imposed upon 1980-2009 climate data, current cultivars and farm management.

Change Factor	**Sensitivity Test Levels**
**[CO_2]**	360, 510, 660, 810 ppm
**ΔT**	-1, 0, +1, +2, +3, +4, +6 ºC
**ΔW**	-50, -30, -20, -10 0, +10, +20, +30%, plus full irrigation
**N**	10, 60, 200 kg/ha

pDSSAT and LPJmL provided all combinations of the simulation, allowing for a simple linear interpolation of yield levels when the HAPPI scenario fell between directly simulated yield levels. Responses are non-linear across the full range of sensitivity tests; however differences between particular sensitivity tests are approximately linear. Nitrogen levels were held constant at current period levels reflecting the high use of fertilizers in North America, Europe, and East Asia compared to lower levels in Latin America and many parts of the developing world. The GEPIC model provided a subset of these simulations (480 sensitivity test combinations), and thus projections were enabled by the use of a mean crop yield emulator:

$$Y = a + b[CO_2] + c(\Delta T) + d(\Delta W) + eN + f[CO_2]^2 + g(T)^2 + h(\Delta W)^2 + iN^2$$
$$+ j[CO_2](\Delta T) + k[CO_2](\Delta W) + l[CO_2]N + m(\Delta T)(\Delta W) + n(\Delta T)N + o(\Delta W)N \qquad \textit{(Eqn. 1)}$$

(a-o) are fit to mean 30-year yields for the 480 GEPIC simulations for each grid cell and crop type. This simplified emulator captures the core system behaviors within the climate change

space evaluated. McDermid et al. (2015) found that similar emulators fit to point-based crop models in the AgMIP Coordinated Climate-Crop Modeling Project (C3MP; Ruane et al., 2014) have low root mean-squared error and high correlations with directly simulated output, although they are likely somewhat conservative in extreme climate changes (e.g., +6 ºC and -50% rainfall). +1.5 and +2.0 ºC Worlds projections rarely extend into these conditions over major agricultural areas. The development of crop yield emulators is a priority of GGCMI and many application communities.

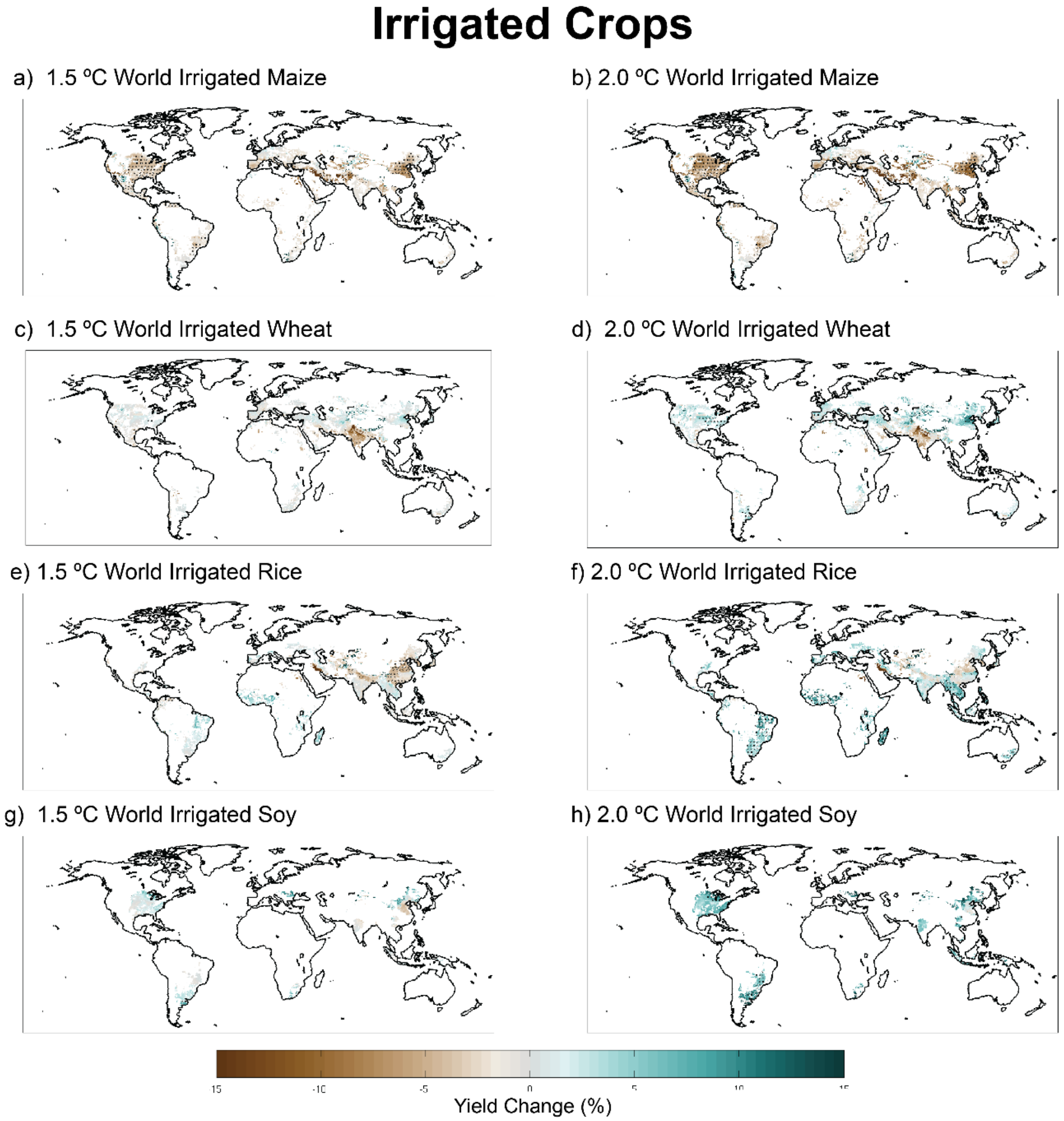

Figure S1: Median yield change projections for irrigated crops across 15 combinations of 5 HAPPI GCMs and 3 GGCMs. Hatch marks indicate regions where 70% of simulations agree on the direction of change. Projections include CO_2 benefits at 423ppm and 487ppm, respectively, for the +1.5 and +2.0 ºC World.
